# Electromyographic and patient-reported outcomes of a computer-guided occlusal adjustment performed on patients suffering from chronic myofascial pain

**DOI:** 10.4317/medoral.20272

**Published:** 2014-12-05

**Authors:** Abraham Dib, Javier Montero, José-Miguel Sanchez, Antonio López-Valverde

**Affiliations:** 1 DDS, PhD. Asociate professor in Dental Prosthesis, Faculty of Medicine, University of Salamanca. Salamanca, Spain; 2 DDS, PhD. Tenured Lecture professor in Dental Prosthesis, Faculty of Medicine, University of Salamanca. Salamanca, Spain; 3 PhD. General Manager Dental School, Faculty of Medicine, University of Salamanca. Salamanca, Spain; 4 MD, PhD. Tenured Lecturer in Periodontics, Faculty of Medicine, University of Salamanca. Salamanca, Spain

## Abstract

Objectives: Muscular hyperactivity is a potential source of symptoms in patients with temporal-mandibular disorders. An adequate occlusal adjustment may relieve such symptoms. This study aims to measure the effect of shortening the protrusive disclusion time (DT) and balancing the center of occlusal forces (COF) on the EMG recordings and assess the pain reported by chronic patients one month after the computer-guided occlusal adjustment. Study Design: The sample studied comprised 34 patients suffering from chronic facial pain in which the EMG activity of both masseters was recorded by electromyography. By selective grinding we alleviated all the occlusal interferences during the mandibular protrusion from the habitual closure position in order to establish an immediate posterior disclusion and an equilibration of the COF. Results: At follow-up 76.5% of the patients reported no facial pain. Moreover, the EMG activity and protrusive DT were significantly reduced, and occlusal and muscular function were significantly more symmetric than at baseline. Conclusions: According to this EMG study, this computer-guided occlusal adjustment is able to reduce the activity of the masseters and the self-reported muscular pain of patients one-month after treatment.

** Key words:**Myofascial pain, occlusal adjustment, electromyography, T-Scan III, occlusal interferences.

## Introduction

Large sections of the populations across the world suffer from temporo-mandibular disorder (TMD). TMD occurs with a dysfunction associated with pain in the muscles of mastication. Accordingly, the symptoms of patients with TMD are musculoskeletal in essence, and mostly derive from long-lasting muscle hyperactivity. Several etiologic factors, such as parafunctional and postural habits, but also psychological and occlusal factors, are usually attributed to the onset of TMD and also to the perpetuation of the muscular-related disturbances. Several authors have reported a strong correlation between occlusal interferences and TMD, based on the rationale that occlusal disturbances lead to mandibular instability, and hence increase the activity of the masticator muscles (for stabilizing the jaw), eventually leading to TMD (1-3).

For about four decades, several authors have indicated the importance of tracking dental occlusion and mandibular movements for the early diagnosis of disturbances of the temporo-mandibular joints (TMJ) (4). In fact, occlusal assessment and rehabilitation are also a permanent challenge in daily dental practice, since dentists’ interventions could alter the usual occlusal scheme of patients with hyper tonic muscles due to fatigue and thus perpetuate or even exacerbate the existing pathology (5). Nevertheless, currently the role of occlusion in TMD is increasingly recognized as a controversial issue between clinicians and researchers (6).

Electromyographic (EMG) recordings allow valid, reproducible and noninvasive measurements of the electrical activity of muscles, which can be made with the muscle both at rest and when active. In this sense, electromyography facilitates the recording of both symptomatic and asymptomatic patients, allowing dentists to monitor bioelectrical changes in the jaw muscles before and after interventions (7). Using EMG assessments, some authors have found a significant reduction in muscle activity when a prolonged disclusion time is shortened to <0.5 sec/excursion with a computer-guided occlusal adjustment (8). One etiological neuromuscular mechanism advanced to explain the association between hyperactivity of the masticator muscles and long disclusion time suggested that the longer the excursive interferences, the longer the periodontal ligaments are compressed, and the longer the masticator muscles are activated to contract (9). The durations of excursive tooth contacts were first measured with the T-Scan instrument (Tekscan Inc., S. Boston, MA) and termed Disclusion Time (10). This was defined as the elapsed time required for a patient to exit from complete intercuspation, and move right, left, or forward, to disclude all of the posterior teeth until only the canines and/or incisors are in contact (10).

The computer-guided occlusal adjustment procedure employed to shorten the pretreatment disclusion time has been termed Complete Anterior Guidance Development Enameloplasty (ICAGDE) (11). This method has been monitored since 1991 (10), and it affords a lasting masticator muscle-relaxing effect and reduces the myalgic symptoms (12,13). In addition to the disclusion time, it has been shown that the presence of unbalanced muscular activities on both sides of the face may be due to asymmetric occlusal contacts during mandibular movements (14), and such functional asymmetry seems to be related to the severity of the signs and symptoms of TMD (15). Accordingly, a coordinated and balanced muscle activity would be an important factor for the correct functioning of the TMJ (16).

Both the disclusion time (DT), and the position of the center of occlusal forces (COF) can be controlled by a computer system (T-Scan III). If this occlusal assessment is combined with a simultaneous EMG recording, it would be possible to check whether the computer-guided selective grinding focusing on shortening the DT and centering the COF is able to reduce the muscular-related pain reported by TMD patients in a cohort follow-up study. This study aimed to measure the effect of shortening the protrusive DT and centering the COF according to both EMG recordings and the pain-discomfort reported by TMD patients one month after the computer-guided occlusal adjustment.

## Material and Methods

This cohort follow-up study was approved by the bioethical committee of the University of Salamanca, all the subjects included in the study being duly informed about the study protocol. Specific written consent was obtained in all cases. The study was conducted with 34 patients with ages ranging between 20 and 60 years, who attended the University Dental Clinic in Salamanca, reporting chronic symptoms of pain-discomfort in the masticator muscles that had been present for the previous three months. The sample was comprised by dental students and faculty staff. All had complete permanent dentition (with the exception of the third molars), with Angle’s class I, and none had systemic pathologies.

At baseline, all participants were asked about the degree of facial discomfort on a Likert scale format (from mild discomfort to very intense pain). Both the electromyographic activity and the dental occlusion were digitally recorded simultaneously in all cases, as done by Kerstein *et al*. (17). A T-Scan III (Tekscan Inc., South Boston, MA, USA) was used to explore the occlusal contacts and determine the COF in maximal intercuspation (MI), and also to record the variation in both parameters when the mandible moved forward until only the anterior teeth occluded (incisors and/or canines). The EMG device (MYOMED_932 ™; Enraf-Nonius B.V., The Netherlands) was employed to record the muscle activity of the masseter bilaterally; it had two channels for continuous monitoring of the activity of the muscles, using 4 self-adhesive electrodes placed bilaterally on the surface area of the masseter, parallel to the direction of the muscle fibers.

For this clinical assessment, the patients sat upright in a dental chair with the back of the chair upright and the head upright, resting on a headrest. Prior to the occlusal assessment, the baseline EMG activity of the masseter was recorded with the lower jaw at rest. Then, the COF was recorded in the MI position and the DT was calculated for protrusion movements as follows. While the dual recording was being made, the patient was asked to close his/her mouth and then begin a protrusive excursion from the MI to the edge-to-edge position at anterior level. Repeated measurements were recorded to verify the reliability of the DT during protrusion. During this process, the fluctuations in the levels of muscle activity of the masseter were recorded simultaneously, continuously and bilaterally to assess dynamic functional activity. However, we only recorded the most representative EMG values of both masseters in the resting position. The recording time in all patients was limited to 2.5 sec.

After these assessment and recordings, a single operator (AD) corrected the occlusal scheme by selective grinding under control of the T-Scan III™ in order to center the COF at the MI position, and shortening the DT to below 0.5 sec in the protrusion movements. The selective grinding was performed according to the following scheme. After drying the superior and inferior occlusal faces, the patient was requested to close his/her mouth in the MI position with an articulating paper (AccuFilm™; Parkell Inc, Edgewood (NY) USA) interposed between the teeth on both sides of the mouth, and then to perform a forward excursion, recording the interferences and then selectively sculpting them to reduce the protrusive DT. The control of the COF was accomplished with the T-Scan III™ by recording the MI and determining points of premature contact. This allowed the possibility of interventions aimed at balancing occlusal forces between both sides of the mouth. This process was repeated until all the marks of the occlusal interferences had disappeared. Finally, the patient was asked to perform several mouth closures in the new MI position, again using the articulating paper. At that moment, the contacts were adjusted, also with selective sculpting, to obtain uniform contacts. Finally, the sculpted teeth were polished. Since we aimed to assess a non-pharmacological approach to miofascial pain, no medication was prescribed. However, all the patients reported they had unsuccessfully taken several drugs in the past for reducing pain and/or relaxing the muscles.

The COF is a quantitative variable that represents the percentage of asymmetry in occlusal loadings, and takes values from 0 (perfect symmetry: bilateral load) to 100 (complete asymmetry: only one-side load). After one month, the dual assessment to control the activity of the masseter and the occlusal forces was repeated and the patients were asked about the presence of muscular pain-discomfort. Two months after the initial procedure, patients were asked again to report about possible facial pain.

-Statistical Analysis

For the statistical analysis, we used parametric and non-parametric tests (Student´s t test and the Chi-Square Test) to compare the quantitative or nominal variables between the two groups respectively. The intra-group longitudinal change was analyzed using paired-T-Tests and McNemar Tests for the comparison of quantitative and nominal variables respectively. The linear association between some quantitative variables was explored using Pearson correlation coefficients (r). The Statistical Package for the Social Sciences v.20. (SPSS Inc., Chicago, IL) was used for the statistical analyses. The cut off level for statistical significance was 0.05.

## Results

The sample mostly comprised females (61.8%), aged 33.1 ± 11.3 years on average, reporting mild to intense muscular pain in the past three months ( Table 1). The data from the statistical analyses revealed that one month after performing the occlusal adjustment, muscular activity and the disclusion time were significantly reduced, and occlusion and muscular function were significantly more symmetric than at baseline ( Table 2). Thus, we observed that after centering the COF, shortening the DT and balancing the muscular function, 76.5% of the patients complaining of distinct degrees of facial pain prior to the adjustment reported a lack of pain at one-month after the occlusal adjustment ( Table 2). There were no significant differences between the pain subgroups regarding either occlusal or muscular parameters, but at baseline patients with residual muscular pain at follow-up tended to have a more deviated CF (26.0±26.2) than those without pain (19.8±16.1). This trend was still evident at follow-up regarding the CF position (3.9±3.2 versus 2.4±1.7). Also, the DT at follow up tended to be greater in the pain subgroup (0.73±0.38 seconds) than in the non-pain subgroup (0.55±0.35 seconds).

**Table 1:**
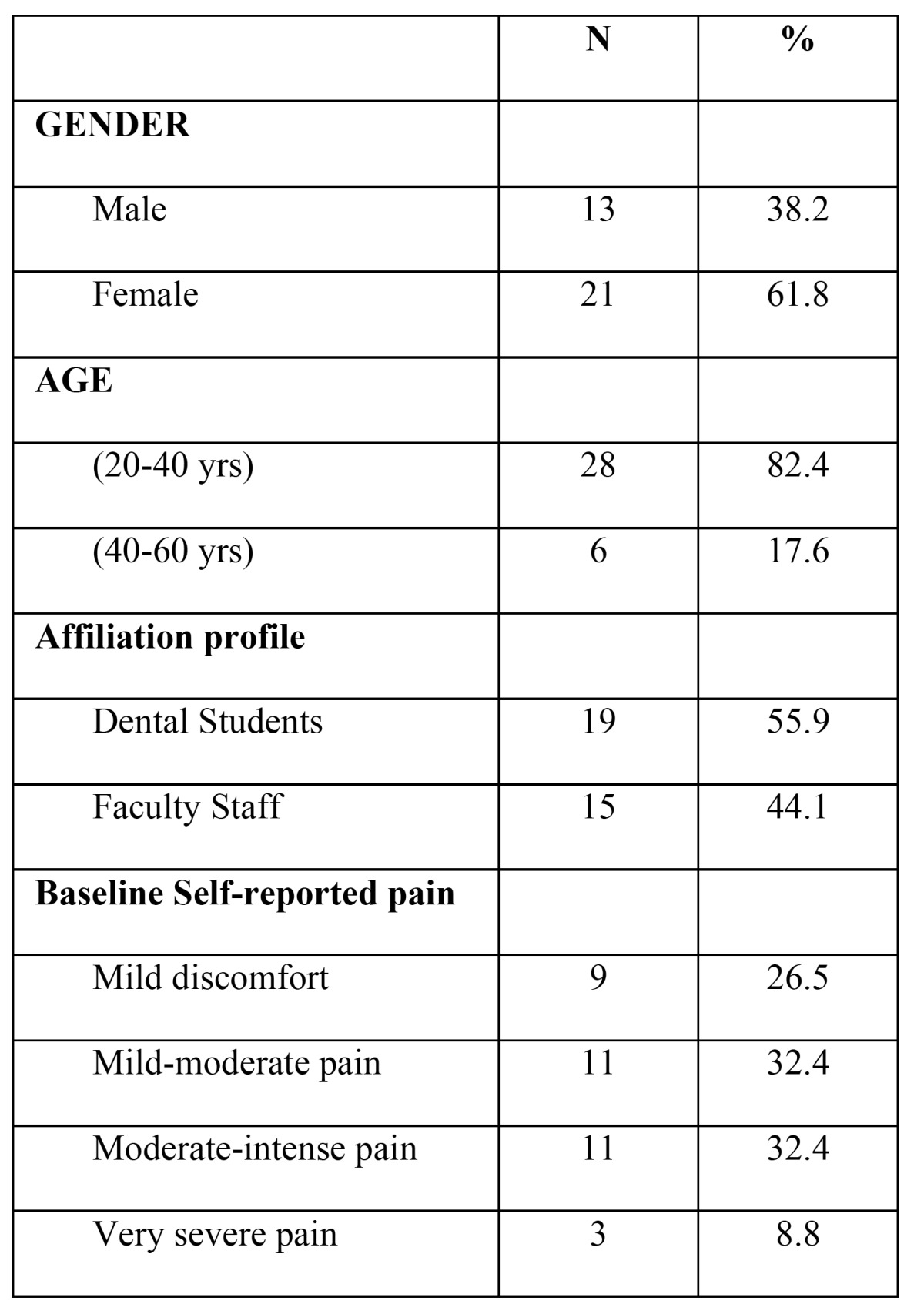
Sociodemographic description of the study sample (n=34), and baseline muscular-related pain.

**Table 2:**
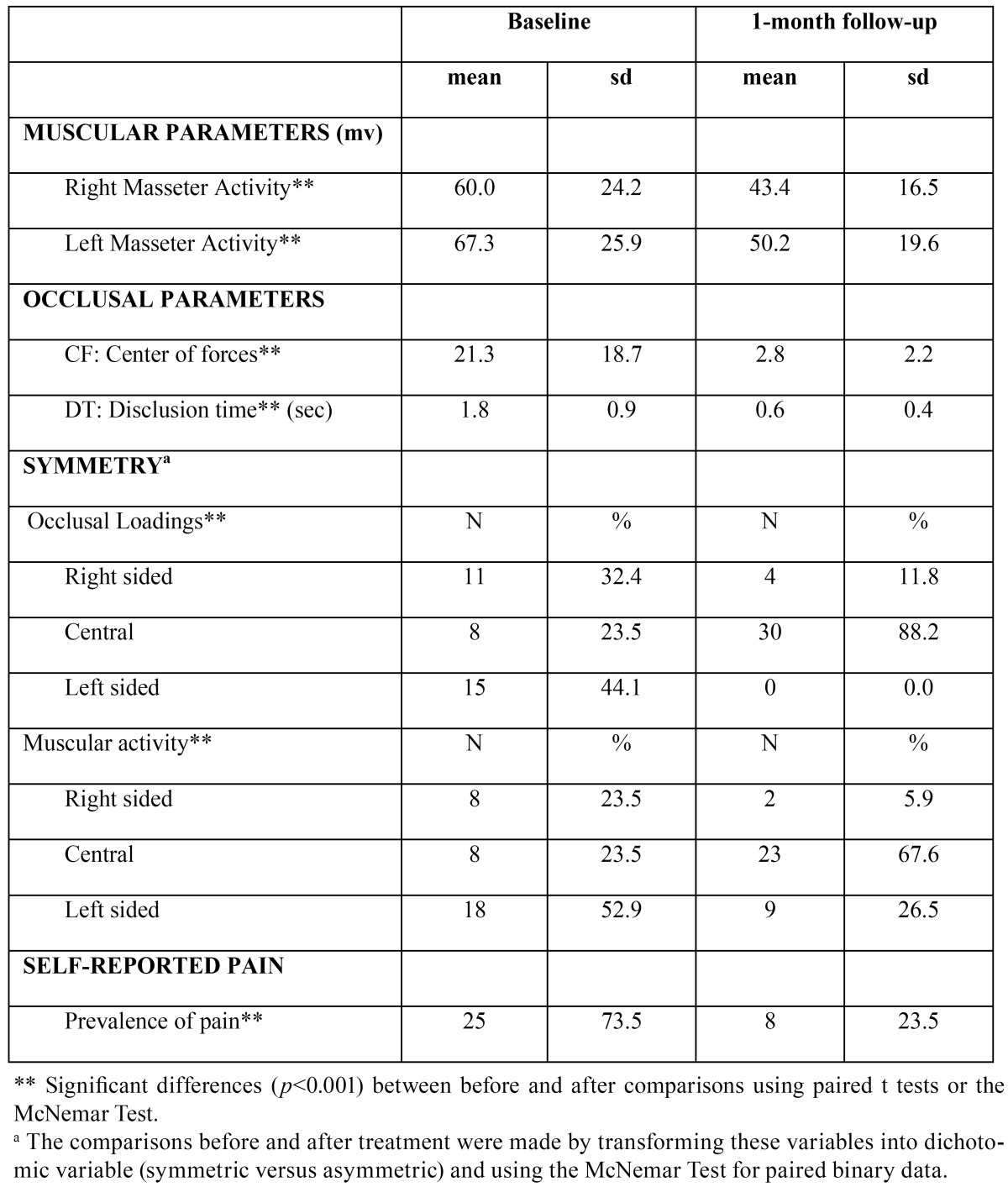
Pretreatment and post-treatment comparisons regarding the muscular and occlusal parameters as well as the prevalence of mild-severe self-reported pain in the study sample (n=34).

After performing correlation tests, and according to the Pearson Correlation Coefficient, we found that the DT was not linearly correlated with the muscular activity or the COF. However, the baseline DT was significantly correlated with the postoperative DT (r=0.5; *p*<0.01), this relationship being clearly higher, and only significant, among females (r= 0.75; *p*<0.001) than among males (r= 0.1; *p*<0.87). Furthermore, in general the baseline DT was significantly correlated with age (r=0.36; *p*<0.05).

Regarding the symmetry parameters, we found that in the follow-up assessment most of the patients with a centered COF also exhibited symmetrical muscular activity (73.3%), whereas those with an asymmetric COF (all right-sided, as depicted  Table 2) in the postoperative assessment also had an asymmetric muscular function (75%), this distribution being significantly different (Chi: 3.8, df:1; *p*<0.05). In addition, the occlusal adjustment seemed to be more effective in the individuals with more preoperative muscular pain than in those reporting only mild muscular discomfort at baseline, since the improvement rate was significantly higher within the former (84%) than in the latter subgroup (55.6%).

 Table 3 depicts the distribution of the sample according to the three clinical goals attained after treatment. Centering of the occlusal forces was the goal most commonly achieved (88.2%) whereas shortening the DT ≤ 0.5 seconds was only achieved in half of the sample (52.9%). In half of the sample, only two out the three goals were achieved, but in 32.4% of the patients all three clinical goals were accomplished. In sum, in 82.4% of the patients an optimal/sub optimal outcome was feasible, and in only 5.9% of the patients were none of the goals reached ( Table 3).

**Table 3:**
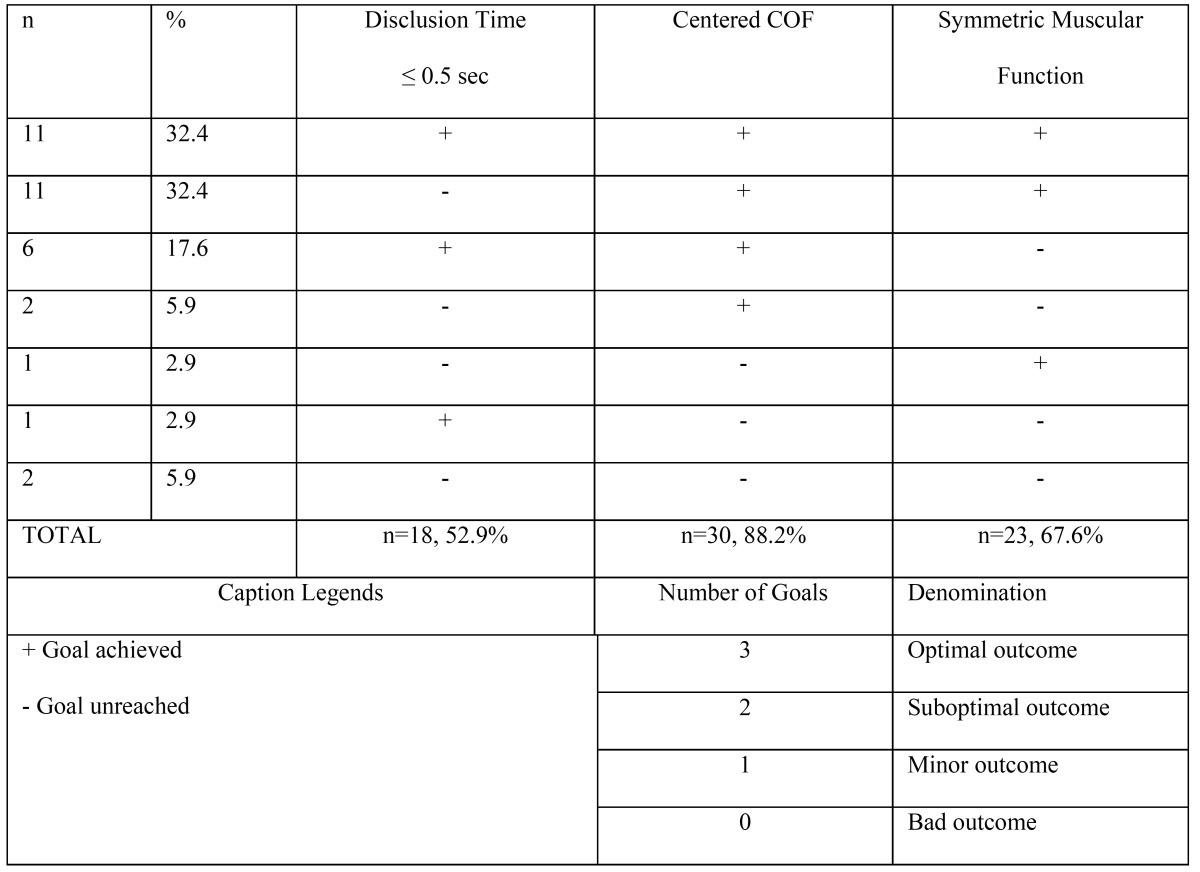
Distribution of the study sample (n=34) according to the goals achieved.

 Table 4 shows the sociodemographic and the clinical characteristics of the patients belonging to the optimal/sub optimal therapeutic subgroups in comparison with the patients in which most of the clinical goals were not achieved (as depicted  Table 3). In terms of the sociodemographic profile of the therapeutic subgroups ( Table 4), age was significantly higher among the optimal/sub optimal subgroup (34.6 ± 11.8 yrs) than their counterparts (26.2 ± 4.3 yrs). The percentage of females within the optimal/sub optimal subgroup tended to be higher than in males, although it was not significant. From the clinical perspective, the muscular activity of the left masseter was significantly lower among the optimal/sub optimal subgroup than their counterparts at baseline and at the follow-up assessment. Furthermore, the reduction in the DT was significantly greater in the optimal/sub optimal subgroup (1.3 ± 0.8 seconds on average within the whole sample). The prevalence of pain tended to be slightly greater at baseline and at follow-up among the Minor/Bad Outcome subgroup.

**Table 4:**
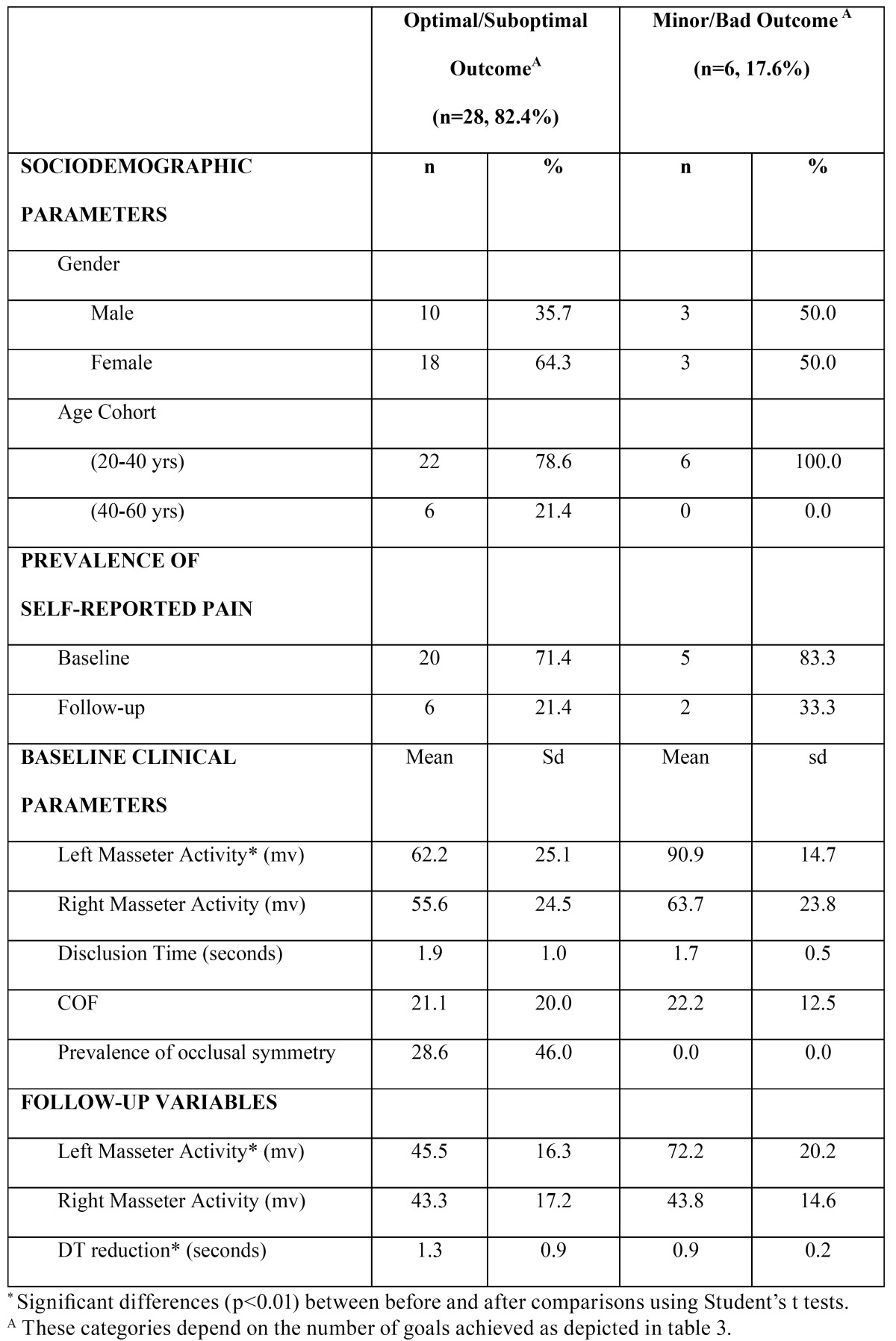
Distribution of certain characteristics of the study sample according to the treatment goals attained (n=34).

The main finding of this study is that one-month after the computer-based adjustment procedure, the percentage of patients free of pain was 76.5%. This finding was accompanied by a reduction in muscular activity and a symmetric function of the masseters. Thus, it seems that centering the COF and shortening the DT improve electromyographic activity and promote the functional symmetry of the masticator muscles, and this could positively influence patient comfort ( Table 3, Table 4).

Two months after the initial procedure, all patients reported that their symptoms had either disappeared completely or were dramatically reduced, and no evidence of muscular hyperactivity was detected on palpation.

## Discussion

Occlusal adjustment has been recommended by different authors to reduce chronic muscular hyperactivity (8-13,17,18) since it can offer efficient treatment for the symptoms of TMJ dysfunction. By contrast, recent systematic reviews have failed to detect any clinical improvements when occlusal adjustment are performed to treat TMD patients (19). The ICAGDE is an occlusal adjustment procedure that requires control over the time and intensity of the occlusal contacts to ensure that the occlusal changes have been correctly accomplished. The traditional procedure based on subjective interpretation of the articulating paper marks is an ineffective clinical method for determining the relative occlusal forces of tooth contacts, and thus a poor guideline for performing occlusal balancing (20).

It could be therefore argued that since most of the evidence regarding the effectiveness of occlusal adjustment for treating and preventing TMD comes from traditional procedures instead of computer-guided adjustments (19), the true effect of a digit ally man aged occlusal balancing is still underreported and underestimated.

This study aimed to evaluate the effect of the ICAGDE on self-reported pain and masseter activity by simultaneously recording the EMG values and the distribution of occlusal contact forces. This intervention was carried out in patients with distinct grades of self-reported pain, because all the patients had previously experienced a refractory effect with the pharmacological approach (pain-killing and miorelaxing drugs). A similar study was carried out by Kerstein in 2006, when 62 patients with orofacial myalgia underwent ICAGDE (17). Kerstein found that one month after the ICAGDE the maximum voluntary contractile strength of both the masseter and temporalis muscles had increased significantly. Moreover, a clear reduction in muscle contraction during excursions was observed as from the time of the first ICAGDE session (18).

However, the findings from Kerstein’s studies should be regarded with caution, because most of them are based on a limited number of patients, mainly during the initial studies in which the samples only comprised seven (10) or six women (12). Furthermore, the 0.5 sec limit for the DT was established empirically by Kerstein, since no ROC curve analyses were made to select the best cutoff point. Even in the most recent study, Kerstein has arbitrarily reduced the DT to 0.4 sec (8) and has ignored the patients’ symptoms, focusing the analysis on the relationship between the DT and EMG recordings, when in fact a patient-centered assessment was the primary goal for the first of Kerstein´s studies (10,21).

Our results highlight the role of the disturbing inputs to the neuromuscular system from the lengthy posterior occlusal contacts during protrusive excursions and the unbalanced occlusal forces in the etiology of chronic myofascial pain in dentate adults. Relief was reported to be immediate, but became more pronounced after a waiting period of about 4-8 weeks. The sequence of occlusal adjustments was focused on establishing an immediate posterior disclusion for mandibular protrusive movements from the habitual closure position of the jaw, without refining the centric relation occlusion.

To our knowledge, since the first ICAGDE study performed in 1991 (10) protrusive excursions have not yet been analyzed or treated. We assumed that lateral excursions are far more prevalent than antero-posterior excursions during masticatory activity, but we believe that treating the protrusive interferences may promote symmetrical muscular activity and thus establish a non-sided masticatory pattern. Furthermore, Kerstein had previously observed that while all his patients had an inability or only limited ability to make right and/or left excursive movements, all were able to protrude their mandibles (21). Thus, our sagittal procedure seems more practical clinically and our procedure is also more pragmatic and more replicable scientifically than the bilateral approach, since we recorded a single DT per patient instead of averaging two commonly asymmetric DTs, as occurs when a bilateral assessment is performed.

In this regard, both the pretreatment and postreatment lateral DT seem to be shorter (10,12,22) than the protrusive DT reported here ( Table 2). Nevertheless, in agreement with Kerstein a reduction of about 1.4 seconds in the DT had a therapeutic effect one month after treatment (18).

We failed to detect any significant correlation between DT and pretreatment masseter activity, as reported by Kerstein in 1991 (10) based on the observations made in 7 women whose EMG and DT recordings did not apparently show any clear trends. Moreover, in ensuing studies by Kerstein, this correlation was not verified. After treatment, on average both the EMG activity and the DT were reduced, but again this concurrent relationship was not linear, nor strong, nor significant. In agreement with our results, Kerstein found a trend towards shorter DTs in women than in men both in patients and in controls, although this difference was not significant (22). The relationship between the activity of the elevator muscles and DT should be further evaluated in large prospective clinical settings.

EMG recordings in the chewing muscles are typically jagged and uneven, but they are also typically asymmetric both when bilateral excursions (8,10) and protrusive excursions ( Table 2) are treated.

One surprising result was that at follow-up the COF deviations tended to be right-sided in 4 patients (11.8%), whereas muscular asymmetry tended to be left-sided (26.5%), as depicted in  Table 2. Thus, occlusal asymmetry seems to be associated with contralateral muscular asymmetry. The balancing of muscular activity is a more challenging goal than centering the COF ( Table 3) which, on the other hand, is an immediate result. These side-related discrepancies have been reported previously in several of Kerstein´s studies (8,10), but no comments/explanations have yet been provided. Under the assumption that this was not a random or spurious finding, a plausible explanation could be that a long standing sided muscular function would always be accompanied by adaptive muscular changes that are still residual one-month later. Such adaptive changes are both anatomical (macroscopically, as hypertrophy, and microscopically as an increase in the number of muscle fiber dimensions, the rate of firing of fibers and the recruitment of additional motor units…) (8), but also postural (a new occlusal relationship would exert a gradual change in neuromuscular control of the masticator muscles). It seems plausible that the waiting period is also necessary for clearing up metabolic residues due to prolonged muscle activity. This might explain why one month later the activity of the left masseter was still slightly higher than that of the right muscle, i.e. the same trend as that observed at baseline.

It is noteworthy that the optimal outcome was only feasible in 1/3 of the patients ( Table 3), it being more common to achieve two out of three therapeutic goals (50%). These findings imply that ICAGDE is a guided but complex clinical procedure that should be performed by experienced clinicians who are aware that the considerable variability in occlusal parameters within patients jeopardizes the achievement of all the goals in most patients treated. The most achievable goal is the centering the COF ( Table 3). Paradoxically, two months after the initial procedure, all patients reported that their symptoms had either disappeared completely or were dramatically reduced. This finding could be the result of a Hawthorne effect, in which the patients responded with the desired outcome, because they appreciate the effort and time spent by the clinician. But also could reveal that our hypothesis is not properly stated, and probably not all the planned goals ( Table 3) have the same influence on the therapeutic effect. Future research with larger sample size should analyze the impact of each goal at a time, to determine the relative effect of each of the planned goals.

Nevertheless, in general our findings support the theory that a reduction in the DT and a balancing of the COF would minimize the compression of the mechanoreceptors of the periodontal ligament and -indirectly with this- the EMG activity of the elevator muscles, thus alleviating the muscle-related facial pain as long as there is still no intrinsic intra-articular pathology (8).

Some limitations of this study should be taken into account. First, we only measured the muscular activity of the masseters, assuming a similar pattern and effect on the temporalis muscles. This decision was made because the masseters were the most prevalently affected muscles in this type of patient, and masseters even seem to be more sensitive to ICAGDE therapy (18). The second limitation of our study is that since we made two EMG assessments in a one-month follow up study, variations due to removing and replacing the EMG electrodes may have occurred, thereby altering the magnitude, but not the direction, of the associations reported here. The results of this occlusal therapy are expected to be stable along time, as reported elsewhere (12,21), further research is certainly necessary. Another strong limitation is the lack of a control group (untreated or sham-treated) in order to control the placebo and other nonspecific effects of the occlusal procedure under consideration. The best study design for assessing the effect of any intervention is the randomized controlled trial. This is especially the case when considering occlusal adjustment for masticatory pain, where the literature has been anything but clear. However the Bioethics Committee of the University of Salamanca could not judged favorably the study protocol, if a group of patients suffering from miofascial pain were not treated with one of the available therapeutic options. Notwithstanding, since ours was a prospective study, before receiving the treatment the subject acted as a control in the paired analyses.

Being aware of the limitations of this study, we concluded that a decrease in the EMG activity of the chewing muscles and patients’ self-reported pain can be achieved one month after a computer-guided occlusal adjustment focusing on balancing the occlusal forces in the usual closure position and shortening the disclusion time during mandibular protrusions.
